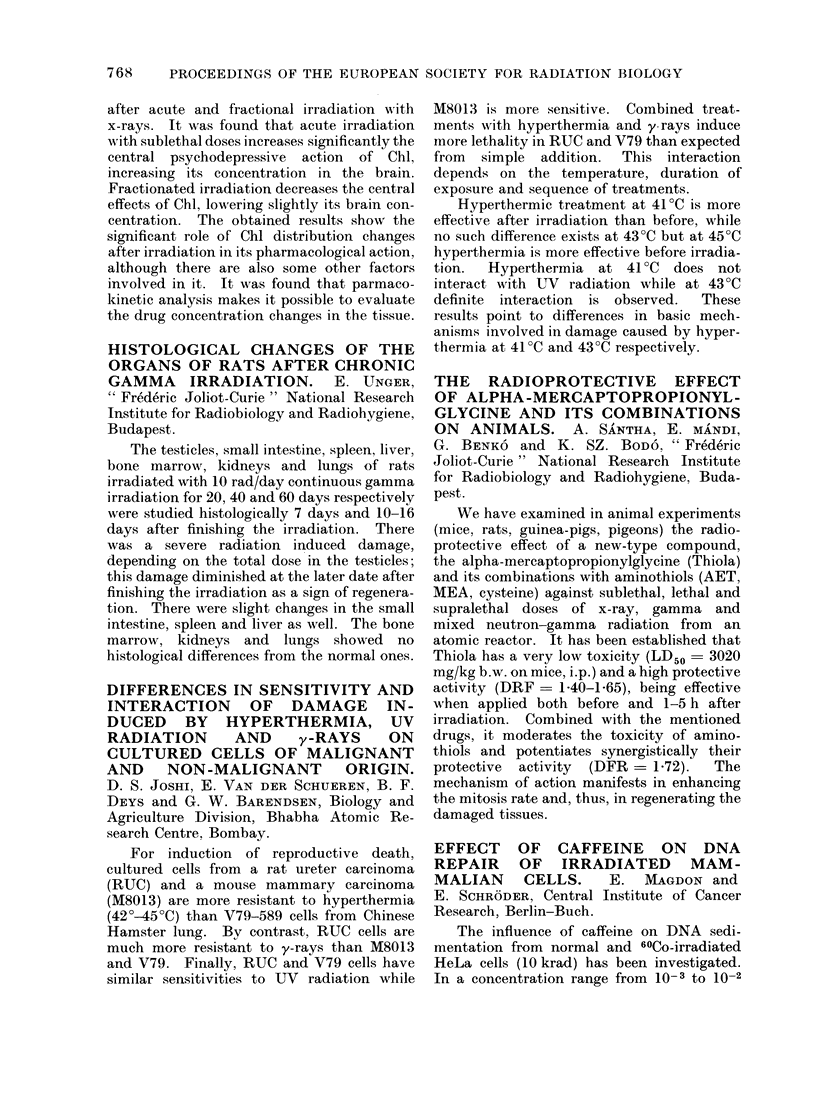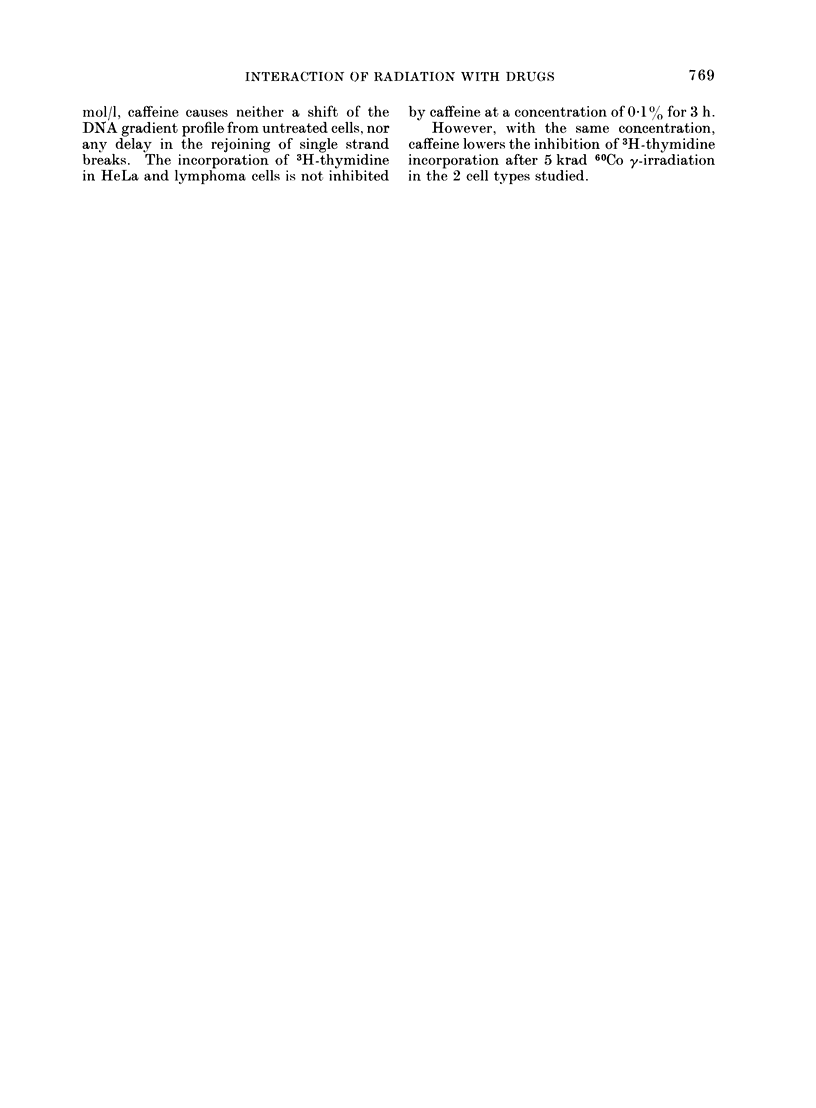# Proceedings: Effect of caffeine on DNA repair of irradiated mammalian cells.

**DOI:** 10.1038/bjc.1975.348

**Published:** 1975-12

**Authors:** E. Magdon, E. Schröder


**EFFECT OF CAFFEINE ON DNA REPAIR OF IRRADIATED MAMMALIAN CELLS.** E. Magdon and E. Schröder, Central Institute of Cancer Research, Berlin–Buch.

The influence of caffeine on DNA sedimentation from normal and $^{60}$Co-irradiated HeLa cells (10 krad) has been investigated. In a concentration range from $10^{-3}$ to $10^{-2}$

mol/l, caffeine causes neither a shift of the DNA gradient profile from untreated cells, nor any delay in the rejoining of single strand breaks. The incorporation of $^{3}H$-thymidine in HeLa and lymphoma cells is not inhibited by caffeine at a concentration of 0·1% for 3 h.

However, with the same concentration, caffeine lowers the inhibition of $^{3}H$-thymidine incorporation after 5 krad $^{60}Co$ $\gamma$-irradiation in the 2 cell types studied.